# Patterns of use of secondary mental health services before and during COVID-19 lockdown: observational study

**DOI:** 10.1192/bjo.2020.104

**Published:** 2020-10-12

**Authors:** Samuel Tromans, Verity Chester, Hannah Harrison, Precina Pankhania, Hanna Booth, Nandini Chakraborty

**Affiliations:** Speciality Registrar in the Psychiatry of Intellectual Disability at the Agnes Unit, Leicestershire Partnership NHS Trust; and an Honorary Academic Clinical Lecturer in the Department of Health Sciences, University of Leicester, UK; Department of Psychiatry, St John's House, Norfolk, UK; and a PhD Student at Norwich Medical School, Norwich, UK; Clinical Studies Officer in the Department of Research and Development, Leicestershire Partnership NHS Trust, UK; Clinical Studies Officer in the Department of Research and Development, Leicestershire Partnership NHS Trust, UK; Speciality Doctor in the Psychosis Intervention and Early Recovery team, Leicestershire Partnership NHS Trust, UK; Psychiatrist in the Psychosis Intervention and Early Recovery team, Leicestershire Partnership NHS Trust, UK

**Keywords:** Epidemiology, inpatient treatment, outpatient treatment, COVID-19, coronavirus

## Abstract

**Background:**

The coronavirus disease 2019 (COVID-19) pandemic has had a profound impact on both the physical and mental well-being of the global population. Relatively few studies have measured the impact of lockdown on utilisation of secondary mental health services in England.

**Aims:**

To describe secondary mental health service utilisation pre-lockdown and during lockdown within Leicestershire, UK, and the numbers of serious incidents during this time frame.

**Method:**

Data pertaining to mental health referral and hospital admissions to adult mental health, child and adolescent mental health, intellectual disability and mental health services for older people were collated retrospectively from electronic records for both 8 weeks pre-lockdown and the first 8 weeks of lockdown in England. Serious incidents during this time frame were also analysed.

**Results:**

Significantly (*P* < 0.05) reduced referrals to a diverse range of mental health services were observed during lockdown, including child and adolescent, adult, older people and intellectual disability services. Although admissions remained relatively stable before and during lockdown for several services, admissions to both acute adult and mental health services for older people were significantly (*P* < 0.05) reduced during lockdown. Numbers of serious incidents in the pre-lockdown and lockdown periods were similar, with 23 incidents pre-lockdown, compared with 20 incidents in lockdown.

**Conclusions:**

To the best of our knowledge, this is the first UK-based study reporting patterns of use of mental health services immediately prior to and during COVID-19 lockdown. Overall numbers of referrals and admissions reduced following commencement of COVID-19 lockdown. Potential reasons for these observations are discussed.

Coronavirus disease 2019 (COVID-19), first recognised in December 2019, has led to a global pandemic of respiratory disease, posing a monumental challenge for public health, clinical research and medical professionals.^1^ As of 13 August 2020, COVID-19 has been confirmed in 20 439 814 people worldwide (313 802 in the UK), and is associated with a mortality rate of approximately 3.6%.^2^ Despite this, findings from England^3^ and China^4^ report reductions in emergency department admissions during COVID-19, with reductions in emergency department consultations of 25% and >50%, respectively. Alongside physical morbidity and mortality, pandemics such as COVID-19 can have a significant impact on the mental health of the affected population.^5^ This may have a consequent effect on the number of referrals to specialist mental health services, as well as the number of admissions to mental health in-patient units. Indeed, a recent French study^6^ reports a 54% reduction in psychiatric emergency consultations in the 4 weeks immediately subsequent to COVID-19 lockdown, compared with the equivalent 4-week period in 2019.

In the UK, the COVID-19 government-regulated lockdown came into immediate effect on the 23 March 2020, following a statement by Prime Minister Boris Johnson.^7^ During the lockdown, UK residents were only permitted to leave their homes for very limited purposes, including shopping for basic necessities, one form of exercise per day, any medical need or to provide care or help a vulnerable person, and travelling to and from work, but only when absolutely necessary. The UK police were granted powers to enforce this lockdown, including via fines and dispersing gatherings. In this paper we report the patterns of both mental health referrals and admissions in one geographical region of the UK in two 8-week periods, immediately prior to lockdown, and immediately following the commencement of lockdown.

## Method

### Participants and procedure

Leicestershire Partnership NHS Trust (LPT) serves around one million people living in both urban and rural areas within Leicester City (approximately 350 000), Leicestershire (approximately 690 000) and Rutland (approximately 40 000).^8^ Compared with England, the population served by LPT has a higher proportion of individuals in their late teens and early twenties, with lower proportions of young children, as well as people in the 30–50 and >70 year age groups. Additionally, the population served by LPT has greater ethnic diversity (with 21.6% of people coming from a Black and minority ethnic background, compared with 14.6% for England), lower rates of disability (with 16.5% of people reporting limitations on their day-to-day activities compared with 17.6% for England), and a slightly higher proportion of married people (48.8%, compared with 46.6% for England).^8^

Retrospective Trust data was collected from electronic case records pertaining to weekly admissions during a 16-week-period from 27 January 2020 to 17 May 2020, corresponding to the 8 weeks immediately prior to (27 January–22 March 2020) and immediately subsequent to (23 March–17 May 2020) COVID-19 government-regulated lockdown in the UK on 23 March 2020 (referred to as the pre-lockdown and lockdown periods, respectively). The COVID-19 lockdown remained consistently in effect throughout the entire lockdown period described in this study.

Data pertaining to admissions, defined as when a patient stayed in a psychiatric hospital for ≥1 night, was collected for adult mental health (AMH) services, including admissions to acute, forensic, psychiatric intensive care (PICU) and rehabilitation beds, yielding an overall AMH admissions total. Similarly, admissions data was collected for both acute and short stay intellectual disability beds, also yielding a total value. Admissions data was also collected for child and adolescent mental health services (CAMHS) and mental health services for older people (MHSOP) beds.

Data for mental health referrals, defined as instances where a patient's care was directed to mental health services, was collected for all the aforementioned psychiatric specialty groups, with AMH data further divided into subgroups, including referrals to community mental health teams, forensic services, the psychosis intervention and early recovery team, urgent triage team, place of safety and the urgent care hub.

Data for the numbers of serious incidents, defined as ‘events in healthcare where the potential for learning is so great, or the consequences to patients, families and carers, staff or organisations are so significant, that they warrant using additional resources to mount a comprehensive response’,^9^ were also collected for the pre-lockdown and lockdown periods.

In order to comply with statistical disclosure standards, where data values within individual cells were less than five, specific values were not reported.

### Ethics

Ethical approval was sought from the Research and Development Service of LPT, who advised that the project was service development and did not require ethical review under the Governance Arrangements for Research Ethics Committees based in the UK.^10^

### Statistical analysis

As a result of the assumptions for parametric analysis not being met, non-parametric tests were used, specifically, the Mann–Whitney *U*-test for comparison of mean weekly admission and referral numbers in the pre-lockdown and lockdown periods for each service type, and for mental health services overall. As the intellectual disability short stay service closed following commencement of lockdown and until the end of the study period, this service type was excluded from the analysis of admissions data. Similarly, as the urgent care hub opened following commencement of lockdown (opening on 6 April 2020), this service was excluded from analysis. As a result of the small numbers of serious incidents, no statistical analysis was performed for this variable, but descriptive data is presented.

## Results

### Admissions

**Fig. 1:**
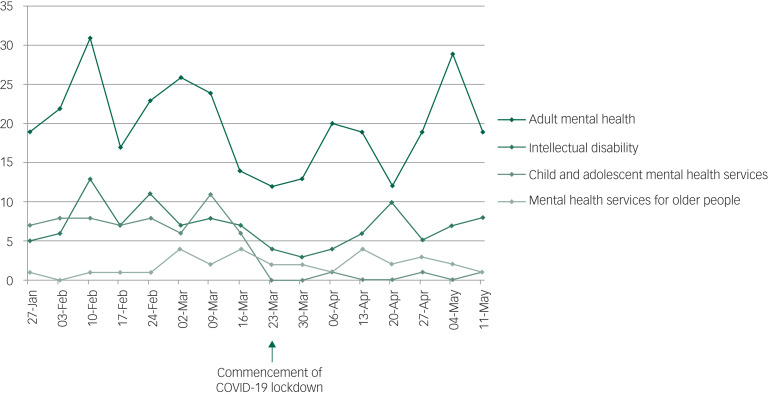
Admissions by service group.

Total admissions to mental health services reduced from 315 in the pre-lockdown period, compared with 210 in the lockdown period. During the lockdown period, lower numbers of admissions were observed for all service types except CAMHS (pre-lockdown *n* = 14; lockdown *n* = 17), PICU (pre-lockdown *n* = 19; lockdown *n* = 20) and intellectual disability acute beds (pre-lockdown *n* ≤ 5; lockdown *n* ≤ 5), where modest increases were observed. Statistically significant (*P* < 0.05) decreases in admissions were observed for acute mental health services for adults (pre-lockdown *n* = 152; lockdown *n* = 121), as well as MHSOP (pre-lockdown *n* = 64; lockdown *n* = 47) (Table 1 and Fig. 1). For raw data pertaining to numbers of admissions on a week-by-week basis, please refer to Supplementary Table 1 available at https://doi.org/10.1192/bjo.2020.104.

**Table 1:** Admissions pre-lockdown and during lockdown

Service type and service subtype	Pre-lockdown admissions				Lockdown admissions				Statistical test		
	*n*^a^	Range^b^	Mean^c^	s.d.	*n*^a^	Range^b^	Mean^c^	s.d.	*U*	*Z*	*P*
Adult mental health											
Acute	152	12–24	19	3.82	121	10–26	15.13	5.00	13.500	−1.683	0.050
Forensic	<5	–	–	–	<5	–	–	–	–	–	ns
Psychiatric intensive care unit	19	1–6	2.38	1.60	20	0–5	2.5	1.85	27.000	−0.119	ns
Rehabilitation	<5	–	–	–	<5	–	–	–	–	–	ns
Adult mental health total	176	14–31	22	5.35	143	12–29	17.88	5.67	14.000	−1.627	ns
Intellectual disability											
Acute	<5	–	–	–	<5	–	–	–	–	–	ns
Short stay	60	6–10	7.5	1.31	N/A	N/A	N/A	N/A	N/A	–	–
Total	61	6–11	7.63	1.60	<5	–	–	–	N/A	–	–
Child and adolescent mental health services	14	0–4	1.75	1.49	17	1–4	2.13	0.99	22.000	−0.723	ns
Mental health services for older people	64	5–13	8	2.67	47	3–10	5.88	2.36	12.500	−1.815	0.040
Overall total	315	39–76	53.88	12.48	210	30–67	44.13	11.97	16.500	−1.337	ns

ns, not significant.

a. Denotes total number of admissions during entire period.

b. Denotes range of number of admissions per week during period.

c. Denotes mean number of admissions per week during period.

### Referrals

**Fig. 2:**
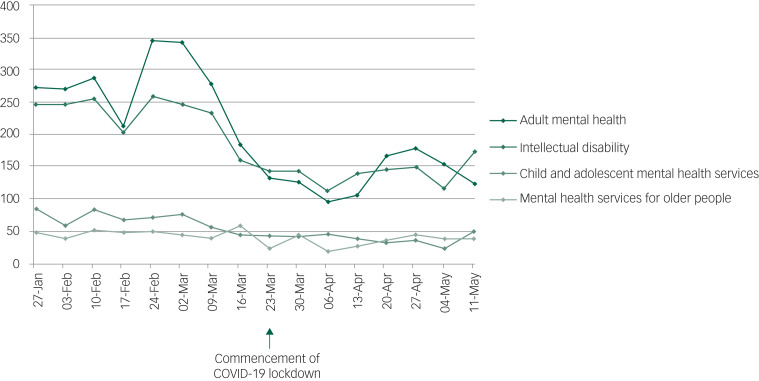
Referrals by service group.

Total referrals to mental health services reduced from 7393 in the pre-lockdown period, to 4622 in the lockdown period. Referrals to all service types within Core AMH reduced significantly (*P* < 0.05), except the forensic service, where there was a slight increase (pre-lockdown *n* = 51; lockdown *n* = 64). Excepting the place of safety service (pre-lockdown *n* = 42; lockdown *n* = 27), all services within AMH saw significant decreases in referrals. Likewise, CAMHS (pre-lockdown *n* = 2193; lockdown *n* = 1081), intellectual disability (pre-lockdown *n* = 539; lockdown *n* = 308), and MHSOP (pre-lockdown *n* = 1850; lockdown *n* = 1120) experienced significantly reduced referrals (Table 2 and Fig. 2). For raw data pertaining to numbers of referrals on a week-by-week basis, please refer to Supplementary Table 2.

**Table 2:** Referrals pre-lockdown and during lockdown

Service type and service subtype	Pre-lockdown referrals				Lockdown referrals				Statistical test		
	*n*^a^	Range^b^	Mean^c^	s.d.	*n*^a^	Range^b^	Mean^c^	s.d.	*U*	*Z*	*P*
Core adult mental health											
Community mental health teams	208	16–39	26	6.85	141	10–31	17.63	7.745	14.500	−1.842	0.034
Psychosis intervention and early recovery	112	8–23	14	5.07	59	3–14	7.38	3.701	9.000	−2.426	0.007
Forensic	51	2–20	6.38	5.88	64	1–16	8	5.372	22.500	−1.007	ns
Core adult mental health, total	371	38–56	46.38	6.21	264	17–45	33	10.226	4.500	−2.894	0.001
Additional adult mental health											
Crisis	1116	116–171	139.5	18.89	723	56–132	90.38	22.608	2.500	−3.100	<0.001
Urgent triage	1282	108–180	160.25	22.81	811	73–123	101.38	16.414	3.000	−3.046	0.001
Place of safety	42	3–8	5.25	1.83	27	1–7	3.38	2.264	17.000	−1.593	ns
Urgent care hub^d^	N/A	N/A	N/A	N/A	288	26–64	48	13.813	N/A	–	–
Child and adolescent mental health services	2193	184–345	274.13	55.85	1081	95–178	135.13	29.059	0.000	−3.361	<0.001
Intellectual disability	539	43–84	67.38	14.00	308	23–50	38.5	8.367	2.000	−3.151	<0.001
Mental health services for older people	1850	159–258	231.25	34.04	1120	112–173	140	19.235	1.000	−3.258	<0.001
Overall total	7393	726–1133	970.5	123.25	4622	469–669	574.75	75.528	0.000	−3.361	0.001

ns, not significant.

a. Denotes total number of admissions during entire period.

b. Denotes range of number of referrals per week during period.

c. Denotes mean number of referrals per week during period.

d. The urgent care hub opened on the 6 April 2020 and was developed with the intention of reducing numbers of patients with mental health problems attending the nearby accident and emergency department of Leicester Royal Infirmary, a hospital nearby.

### Serious incidents

There were a total of 23 serious incidents in the pre-lockdown period, including 11 in the community setting and 12 in the in-patient hospital setting. This compares with 20 serious incidents in the lockdown period, including 12 in the community setting and 8 in the in-patient hospital setting. Please note that individual numbers of serious incidents within psychiatric subspecialties have not been reported in order to comply with statistical disclosure standards.

## Discussion

This paper reports the numbers of referrals and admissions to mental health services in a large geographical area within England in the weeks immediately preceding and during COVID-19 lockdown. Although significant (*P* < 0.05) reductions in referrals to mental health services were observed across diverse clinical populations and age groups during the lockdown period, admissions remained relatively stable to several services, with the exception of those to acute mental health services for adults (pre-lockdown *n* = 152; lockdown *n* = 121), and MHSOP (pre-lockdown *n* = 64; lockdown *n* = 47), both of which decreased significantly (*P* < 0.05). The reduced admissions to acute mental health services for adults should be interpreted with some caution, however, as shortly after commencement of lockdown, one of the seven acute mental health adult wards (a 20-bedded ward) was temporarily converted to a CAMHS ward, as the previous CAMHS ward being used pre-lockdown, based at a separate hospital site, was closed.

Few changes were observed in the numbers of serious incidents during this time (pre-lockdown *n* = 23; lockdown *n* = 20). Although one may have suspected that reduced numbers of referrals and admissions would have reflected in increased crisis situations and related serious untoward incidents, this was not observed in our study. There are several potential explanations for reduced referrals and admissions to secondary mental health services. It is unclear whether this is attributable to patient factors (such as fewer patients seeking mental health support) or to healthcare factors (such as patients seeking support and not being referred/admitted to secondary healthcare), or a combination of the two.

### Healthcare factors

Regarding healthcare factors, it is possible the referral reduction could reflect a reduced healthcare workforce, because of staff self-isolating or being on sickness absence leave with COVID-19 symptoms.^11^ Public health messaging has encouraged patients to avoid immediately attending emergency departments if possible,^12^ which usually provide a major entry point for the mental healthcare system,^13^ and data from both England and China demonstrate substantial reductions in emergency department attendance. Furthermore, when patients do present at emergency departments, delays in mental health assessments and psychiatric admissions may occur as a consequence of requiring confirmation of negative COVID-19 test results.^13^ Equally, admission-related decisions made by mental health professionals may have been altered by COVID-19-related concerns, resulting in a heightened clinical threshold for deciding to admit a patient. It is also conceivable that some gatekeepers may have believed certain in-patient mental health services were closed during lockdown. Indeed, in the Trust in which the present study is based, one service did close during lockdown, the short stay unit service for patients with intellectual disability.

### Patient factors

Reduced referrals could reflect reluctance from patients to present to referral sources, such as the general practitioner or emergency departments. Reduced attendance could also relate to fears of being admitted to hospital and contracting COVID-19,^14^ and a desire to stay in their usual place of residence rather than a group ward with other patients and staff.^15^ Reports of personal protective equipment shortages within healthcare settings may have further served to increase such concerns.^16^ Reduced attendance could be attributed to patients’ not wishing to be a burden or not perceiving their needs as worthy of clinical attention during this critical time for health services,^17^ because of self-isolation, or shielding as a result of increased rates of physical comorbidities among those with serious mental illnesses. Related to this, research from Italy found a significantly reduced rate of acute coronary syndrome (ACS)-related hospital admissions during the COVID-19 pandemic, suggesting that some patients may have died from ACS without seeking medical attention.^17^

It is also possible that the psychiatric needs of some patient's were somewhat ameliorated during COVID-19 lockdown. A developing body of research is suggested that for certain groups, there has been an unexpected increase in well-being during COVID-19, known as the ‘lockdown paradox’.^18^ In France, Pignon and colleagues^6^ reported a 54% drop in psychiatric emergency consultations in the first 4 weeks of COVID-19 lockdown, relative to the same period in 2019. The authors cited possible increased strengths and improved coping strategies during disasters as a possible explanation for this phenomenon,^16^ as such a trend in mental health presentations was also observed following the September 11 World Trade Centre terrorist attacks.^19^

As many people with psychiatric needs rely on members of their support network providing a caring role in order to function, it is possible that the public health messaging of ‘stay at home’^20^ and the government scheme of furloughing employees^21^ may have facilitated the availability of increased social/familial support within the household. Another protective factor could be reduced societal pressure, such as reduced interpersonal stressors because of working from home.^18^ Furthermore, charitable involvement during COVID-19, including increased online mental health resources,^22^ as well as provision of food packages to vulnerable people by the government^23^ could have exerted additional protective effects on mental health and well-being.

### Combination of patient/healthcare factors

Conversely, research has indicated that mental health needs are increasing among certain individuals and groups, such as those experiencing risk factors such as financial stress, occupational instability^24^ and working in healthcare professional roles.^11^ Social distancing may limit opportunities to obtain support from friends and family members, potentially causing loneliness.^25^ Anxiety around contracting the virus may be particularly prevalent among vulnerable groups, such as those with compromised immune function, older persons and Black and minority ethnic populations, where their increased risk has been well publicised.^16^ However, although overall anxiety levels may have increased among the general population in the wake of COVID-19, much of this anxiety may be subsyndromal in severity, rather than representing severe mental illness requiring urgent medical attention.^26^ Patients may feel obligated to abide by government directives to stay at home, even if feeling significantly unwell.^16^

### Limitations

The data reported here is from a single healthcare trust in England, and thus may not be generalisable to all regions. Unfortunately, it was not possible to examine the sociodemographic or clinical factors of patients referred or admitted. It might be considered that patients being admitted to mental health services are those with higher or immediate needs; however, inferences regarding such characteristics cannot be drawn from this data. The study reports data in the weeks immediately preceding and following commencement of COVID-19 lockdown within the UK and may not generalise to service utilisation in the longer-term course of the virus’ trajectory. Nevertheless, the findings provide several interesting observations that could have implications for the response to the current and future pandemics and for future research.

### Clinical implications

Regarding the current COVID-19 pandemic, as well as potential future pandemics, the results suggest that a practical strategy of informing service gatekeepers about which secondary mental health services remain open, and any temporary alterations to usual referral processes could be of benefit. Similarly, this could be coupled with a patient-facing public health strategy, informing them of symptoms for which they should seek primary or secondary healthcare support and about ways to maintain good mental health during a pandemic. Use of telemedicine for supporting patients with mental health problems, which has sharply increased in response to COVID-19,^27^ shows both therapeutic promise^28^ and excellent rates of acceptability from both patients and psychiatrists,^29^ but there is a need for further research as to who this could work most effectively for, as such an approach may be less suitable for certain groups, such as individuals with intellectual disability and/or those lacking access to the required technology.^30^

### Research implications

Future research is required to elucidate the medium–long-term impact of COVID-19 and lockdown on mental health patients and their patterns of service utilisation. This research should incorporate the sociodemographic and clinical characteristics of patients, such as age, gender, ethnicity and whether the patient has a history of engagement with psychiatric services. Medium- and long-term studies could provide insight into whether the mental health impact of the pandemic is more delayed rather than immediately observable. Qualitative research could provide an insight into the perspectives of those with psychiatric disorders and their support network regarding their personal reasons for presenting to services or otherwise, or their thoughts regarding their likelihood of being referred/admitted to secondary services from primary healthcare settings. This would help to inform public health strategies and healthcare resource planning and help to ensure mental health services are available for individuals and populations that require them.

## Data Availability

The data that support the findings of this study are available from the corresponding author, N.C., upon reasonable request.

## Supplementary material

For supplementary material accompanying this paper visit http://dx.doi.org/10.1192/bjo.2020.104. click here to view supplementary material
