# Supplementary material for: Patterns of use of secondary mental health services before and during COVID-19 lockdown: observational study
Source: BJPsych Open. 2020 Oct 12;6(6):e117. doi: 10.1192/bjo.2020.104 (PMC7550872; doi:10.1192/bjo.2020.104)
Supplement: Supplementary file 1 [file bjosup.zip › S2056472420001040sup001.docx]

## Supplementary Table 1 Admissions

| **Week** | **Adult Mental Health** | | | | | **Intellectual Disability** | | | **CAMHS** | **MHSOP** | **Overall Total** |
| --- | --- | --- | --- | --- | --- | --- | --- | --- | --- | --- | --- |
|  |  |  |  |  |  |  |  |  |  |  |  |
|  | **Acute** | **Forensic** | **PICU** | **Rehabilitation** | **AMH**  **Total** | **Acute** | **Short**  **Stay^[[1]](#footnote-1)^** | **ID**  **T**  **otal** |  |  |  |
|  |  |  |  |  |  |  |  |  |  |  |  |
| **27-Jan** | 18 | <5 | <5 | <5 | 19 | <5 | 7 | 7 | <5 | 5 | **32** |
| **03-Feb** | 19 | <5 | <5 | <5 | 22 | <5 | 8 | 8 | <5 | 6 | **36** |
| **10-Feb** | 24 | <5 | 6 | <5 | 31 | <5 | 8 | 8 | <5 | 13 | **53** |
| **17-Feb** | 16 | <5 | <5 | <5 | 17 | <5 | 7 | 7 | <5 | 7 | **32** |
| **24-Feb** | 20 | <5 | <5 | <5 | 23 | <5 | 8 | 8 | <5 | 11 | **43** |
| **02-Mar** | 23 | <5 | <5 | <5 | 26 | <5 | 6 | 6 | <5 | 7 | **43** |
| **09-Mar** | 20 | <5 | <5 | <5 | 24 | <5 | 10 | 11 | <5 | 8 | **45** |
| **16-Mar** | 12 | <5 | <5 | <5 | 14 | <5 | 6 | 6 | <5 | 7 | **31** |
| **23-Mar** | 10 | <5 | <5 | <5 | 12 | <5 | 0 | <5 | <5 | <5 | **18** |
| **30-Mar** | 13 | <5 | <5 | <5 | 13 | <5 | 0 | <5 | <5 | <5 | **18** |
| **06-Apr** | 15 | <5 | 5 | <5 | 20 | <5 | 0 | <5 | <5 | <5 | **26** |
| **13-Apr** | 16 | <5 | <5 | <5 | 19 | <5 | 0 | <5 | <5 | 6 | **29** |
| **20-Apr** | 11 | <5 | <5 | <5 | 12 | <5 | 0 | <5 | <5 | 10 | **24** |
| **27-Apr** | 17 | <5 | <5 | <5 | 19 | <5 | 0 | <5 | <5 | 5 | **28** |
| **04-May** | 26 | <5 | <5 | <5 | 29 | <5 | 0 | <5 | <5 | 7 | **38** |
| **11-May** | 13 | <5 | 5 | <5 | 19 | <5 | 0 | <5 | <5 | 8 | **29** |
| **Total** | **273** | <5 | **39** | <5 | **319** | <5 | **60** | **64** | **31** | **111** | **525** |

1. The ID short stay units closed following commencement of lockdown and remained closed until the end of the study period. [↑](#footnote-ref-1)
