# Supplementary material for: Patterns of use of secondary mental health services before and during COVID-19 lockdown: observational study
Source: BJPsych Open. 2020 Oct 12;6(6):e117. doi: 10.1192/bjo.2020.104 (PMC7550872; doi:10.1192/bjo.2020.104)
Supplement: Supplementary file 1 [file bjosup.zip › S2056472420001040sup002.docx]

## Supplementary Table 2 Referrals

| **Week** | **Adult Mental Health** | | | | | | | | **CAMHS** | **Intellectual**  **Disability** | **MHSOP** | **Total** |
| --- | --- | --- | --- | --- | --- | --- | --- | --- | --- | --- | --- | --- |
|  | **Core AMH** | | | | **Additional AMH** | | | |  |  |  |  |
|  | **CMHT** | **PIER** | **Forensic** | **Core AMH Total** | **Crisis** | **Urgent Triage** | **Place of Safety** | **Urgent Care Hub^[[1]](#footnote-1)^** |  |  |  |  |
| **27-Jan** | 30 | 13 | <5 | 47 | 122 | 176 | 6 | N/A | 273 | 84 | 249 | **957** |
| **03-Feb** | 24 | 10 | <5 | 38 | 132 | 165 | 6 | N/A | 271 | 59 | 247 | **918** |
| **10-Feb** | 25 | 23 | <5 | 51 | 134 | 157 | <5 | N/A | 288 | 82 | 255 | **970** |
| **17-Feb** | 16 | 10 | 20 | 46 | 161 | 172 | 5 | N/A | 212 | 68 | 203 | **867** |
| **24-Feb** | 29 | 19 | <5 | 50 | 171 | 180 | 8 | N/A | 345 | 71 | 258 | **1083** |
| **02-Mar** | 24 | 16 | 5 | 45 | 147 | 169 | 7 | N/A | 342 | 76 | 247 | **1033** |
| **09-Mar** | 21 | 8 | 9 | 38 | 133 | 155 | <5 | N/A | 278 | 56 | 232 | **895** |
| **16-Mar** | 39 | 13 | <5 | 56 | 116 | 108 | <5 | N/A | 184 | 43 | 159 | **670** |
| **23-Mar** | 10 | 6 | 6 | 22 | 56 | 73 | <5 | N/A | 131 | 42 | 142 | **469** |
| **30-Mar** | 31 | 8 | 6 | 45 | 77 | 88 | <5 | N/A | 127 | 41 | 144 | **523** |
| **06-Apr** | 11 | 5 | <5 | 17 | 74 | 101 | 7 | 26 | 95 | 46 | 112 | **478** |
| **13-Apr** | 11 | 11 | <5 | 26 | 90 | 98 | <5 | 46 | 105 | 38 | 138 | **542** |
| **20-Apr** | 25 | <5 | 7 | 36 | 132 | 97 | <5 | 53 | 166 | 33 | 146 | **667** |
| **27-Apr** | 22 | 14 | 8 | 44 | 97 | 117 | 5 | 40 | 178 | 35 | 149 | **665** |
| **04-May** | 18 | <5 | 16 | 37 | 94 | 123 | 5 | 64 | 155 | 23 | 116 | **617** |
| **11-May** | 13 | 8 | 16 | 37 | 103 | 114 | <5 | 59 | 124 | 50 | 173 | **661** |
| **Total** | **349** | **171** | **115** | 635 | **1839** | **2093** | **69** | **288** | **3274** | **847** | **2970** | **12015** |

1. The urgent care hub opened on the 6^th^ April and was developed with the intention of reducing patients with mental health problems attending at the Accident and Emergency department of Leicester Royal Infirmary. [↑](#footnote-ref-1)
